# Determinants of low Apgar score among newborns delivered by Cesarean section in Nigist Eleni Mohammed memorial specialized hospital, Southern Ethiopia

**DOI:** 10.1038/s41598-024-62223-8

**Published:** 2024-05-30

**Authors:** Mitiku Desalegn, Tadele Yohannes, Legesse Tesfaye

**Affiliations:** 1 Department of Anesthesia, College of Medicine and Health Sciences, Wachemo University, Hosaina, Ethiopia; 2 School of Public Health, College of Medicine & Health Sciences, Wachemo University, Hosaina, Ethiopia

**Keywords:** Apgar score, Fifth-minute, Determinants, Case–control, Southern Ethiopia, Epidemiology, Paediatric research

## Abstract

A variety of factors can predispose newborns to have a low Apgar score after delivery. Identification of the determinants of low Apgar scores is an important first step to take to apply the necessary precautions. This study aimed to identify the determinants of low fifth-minute Apgar score after a Cesarean section. An institutional-based case–control study was conducted among mothers who deliver their newborns by Cesarean section in Nigist Eleni Mohammed Memorial Comprehensive Specialized Hospital, Ethiopia, from July 1, 2022, to September 30, 2022. Data were collected from 70 cases and 140 controls using a semi-structured checklist. A systematic random sampling technique was used to select both charts of mothers with cases and controls. Charts of mothers with newborns Apgar score less than 7 were considered as cases; whereas a similar group of charts of mothers with newborns with fifth-minute Apgar score greater than or equal to 7 were categorized as control. Descriptive statistics and bivariable and multivariable binary logistic regression analyses were conducted to describe the mothers and newborns and identify determinants of the fifth-minute low Apgar score, respectively. Adjusted odds ratios (AOR) with their respective 95% confidence interval (CI) were used to declare the determinant factors, and the statistical significance was set at P < 0.05. In total, 140 controls and 70 cases of mothers charts were enrolled in this study. The Mean ± SD age of mothers of cases and controls were 26.9 ± 4.9 and 27.06 ± 4.1 years, respectively. General anaesthesia (AOR = 4.2; 95% CI: 1.9 ‒ 9.3), rural residence (AOR = 3.7, 95% CI, 1.7‒8.1), low birth weight (AOR = 3.2, 95% CI, 1.3‒7.8), and emergency Cesarean section (AOR = 2.6; 95% CI: 1.2 ‒ 5.8) were identified determinant factors of low fifth minute Apgar score. A fifth-minute low Apgar score was significantly associated with newborns delivered through emergency Cesarean section, low birth weight, rural residence, and delivered from mothers who had undergone Cesarean section under general anaesthesia.

## Introduction

The Apgar score was developed in 1952 as a quick way to examine the clinical condition of newborns and is still widely used for newborn infant assessment at 1 and 5 min after birth^1^. It comprises five components: heart rate, respiratory rate, reflex irritability, skin colour, and muscle tone. Each item’s score ranges from 0 to 2, with a total score of 10. A score ≥ 7 requires only routine newborn care. A score between 4 and 6 requires some intervention, whereas a score ≤ 4 requires extensive resuscitation^2^.

Common neonatal complications following Cesarean section include a low Apgar score, perinatal asphyxia, neonatal sepsis, meconium aspiration syndrome, early neonatal death, stillbirth, and prematurity^3^. Newborns with low Apgar scores have a poor neonatal outcome, often with impaired gas exchange, which can escalate to hypercapnia, hypoxemia, and severe metabolic acidosis if left untreated. These conditions cause 24% of infant deaths in Africa, and 280,000 neonatal deaths in Sub-Saharan Africa are attributable to this condition^4^. Infants with a low 5 min Apgar score have a greater risk of mortality and morbidity compared to infants with a low 1 min Apgar score. Of infants with a very low Apgar score at 5 min, 81% had a poor outcome^5^. Fifth-minute low Apgar score survival is a major determinant of a newborn's capacity to thrive; therefore, it is a better predictor of survival than the first-minute low Apgar score^6^. According to recent data from South Australia, children who have five-minute Apgar scores of 0–5 or 6, compared with those of 10, are more likely to achieve at or below the National Minimum Standards (NMS) on the Australian National Assessment Program—Literacy and Numeracy (NAPLAN) assessments when they are eight years old^7^.

A variety of conditions can predispose newborns to have a low Apgar score after a Cesarean section. A comprehensive population study conducted in Sweden found that a more prolonged second stage of labour is linked to a greater likelihood of a low 5-min Apgar score^8^. Low Apgar scores and neonatal complications were shown to occur two to three times more often in emergency surgical deliveries than in elective procedures, according to another Norwegian study^9^. An Apgar score of less than five at five minutes is a reliable indicator of newborn mortality in infants weighing between 1500 and 2499 g at birth^10^. Data from Ethiopian settings show that the following variables were independently associated with the low Apgar score: type of anaesthesia, meconium-stained amniotic fluid, antepartum haemorrhage, skin incision to delivery time, pregnancy-induced hypertension, and fetal birth weight < 2.5 kg^11^.

In studies performed in several countries, birth asphyxia among babies born by Cesarean sections is associated as evidenced by low Apgar scores. Determinant factors associated with low fifth-minute Apgar scores on newborns with Cesarean section have not been studied well in Ethiopia. Identification of the factors associated with low fifth-minute Apgar score after Cesarean section is an important first step for clinicians for the management of such a group of pregnant women to take the necessary precautions. This will reduce neonatal morbidity and mortality significantly and will help to meet the 2030 SDG (Sustainable Development Goal) goal of the country. Other researchers in this area also can use this paper as input to research the practice of Cesarean section. The objective of this study was to identify the determinants of low fifth-minute Apgar score in newborns delivered by Cesarean section at the Nigist Eleni Mohamed Memorial Comprehensive Specialized Hospital.

## Method and material

### Study design

This was an institution-based, unmatched and retrospective case–control study.

### Setting and study period

The study was conducted at Wachemo University Nigist Eleni Mohammed Memorial Comprehensive Specialized Hospital from July 1, 2022, to September 30, 2022. It is located in Hossana town, Hadya Zone, South Nation’s Nationalities and Peoples Region 232 km, south of Addis Ababa, the capital city of Ethiopia. The maternity ward has 80 beds, on average; there are 30 deliveries each day. From the total number, One-fourth of newborns were delivered by cesarean section. Spinal anaesthesia is the most widely practised type of anaesthesia for Cesarean sections, and most of the cases indicated to undergo Cesarean section are emergencies. Three nurse midwives in the operating theatre work interchangeably for the entire 24 h of a day in an 8-h shift, along with seven obstetrics and gynaecology specialists and 12 anaesthetists. The assigned nurse-midwives are trained to perform newborn care. In case of a neonatal emergency, the gynaecologists and master anesthetists provide advanced neonatal resuscitation together.

### Participants

All charts of mothers with term or post-term neonates who gave birth with Cesarian section were included in the study. Charts of mothers with, multiple pregnancies, neonates with congenital anomalies, intrauterine fetal death diagnosed on admission, whose mothers were unable to provide a medical history due to any medical disease or obstetric complications, and neonates from a mother with medical illnesses (obesity, diabetes mellitus, cardiac illness and asthma) were excluded from the study.

### Sample size determination and sampling technique

The sample size was calculated using the double population proportion formula by using Open Epi info for an unmatched case–control study. The proportion of exposure among controls and cases of different variables, which were stated in different literature as a determinant factor of a low Apgar score, was computed. From the listed alternative sample size, the largest sample size was selected (Table 1). The following assumptions were used; 95% confidence level, power of 80%, the OR (odds ratio) of 4.58, proportion (p) of controls with preeclampsia of 5.9%^11^, and 2:1 controls to cases ratio, the final sample size was (210; 70 cases and 140 controls).

**Table 1:** Sample size calculation for unmatched case–control study by open Epi software.

No	Variables	Proportion of controls with exposure	Proportion of cases with exposure	95%CI OR	Sample size	Ref
1	Induction	59.3%	87.6%	4.85(1.71, 13)	Cases: 34 Controls: 68	^12^
2	I-D time	4.6%	23.6%	5.27(2.2, 12.6)	Cases:32 Controls: 64	^11^
3	Preeclampsia	5.9%	18.2%	4.58(1.7‒11.9)	Cases: 70 Controls: 140	^11^

Before selecting cases and controls, all eligible charts of mothers who underwent Cesarean section during the study period were identified and the data collectors reviewed the identified charts of mothers. From the chart, the Apgar score of every newborn was checked, and charts of mothers with newborns who delivered with a fifth-minute Apgar score < 7 were considered cases, whereas a similar group of charts of mothers with newborns with a fifth-minute Apgar score ≥ 7 was categorized as control. The total number of eligible charts of mothers identified during the study period was 595 (146 cases and 449 controls) then, a systematic random sampling technique was used to select both cases and controls. For both cases and controls, K was determined using the formula: K = N/n, where n = total calculated sample size of cases or controls and N = total identified charts of cases or controls. Therefore, the sampling interval (k) was two 2 for cases and three 3 for controls; the first chart was selected randomly.

### Operational definition

**Cases:** Charts of mothers with newborns with an Apgar score < 7 at the fifth minute following cesarean section delivery. **Controls:** Charts of the mother with a newborn with an Apgar score 7–10 at the fifth minute following cesarean section delivery^13^.

**Low birth weight:** Birth weight of newborn ≤ 2500 g. **Normal birth weight:** Birth weight of newborn > 2500 g.

**Incision to delivery time (I-D time):** the time interval, in minutes, between when the obstetrician makes the skin incision to the delivery of the foetus from the womb.

### Data collection and variables

A semi-structured checklist was used to collect data by reviewing the mother’s charts retrospectively. The checklist was developed after reviewing the literature and mainly addressed the socio-demographic variables of mothers (age, residence); maternal characteristics (parity, antenatal care (ANC) follow-up, anaemia); neonatal characteristics (weight of newborn, outcome of the newborn), and operation-related characteristics ( indication for Cesarean section, Urgency of surgery, type of anaesthesia and incision to delivery time (ID)).

Three midwives are assigned to collect data in an eight-hour shift for 24 h after appropriate training and orientation. Charts of mothers who fulfilled the eligibility criteria were reviewed for relevant information. The principal investigator checked the completeness of the data every day. The authors do not have access to information that could identify individual mothers during or after data collection.

### Statistical methods

The data were coded, entered into Epi Info version 7, and exported to the Statistical Package for Social Sciences (SPSS) software version 27.0. Descriptive statistical analysis was performed to compute the frequency, percentage, and mean of socio-demographic variables. The Apgar score was coded as ''0'' for a low Apgar score and ''1'' for a good Apgar score. When smaller expected frequencies were encountered, re-categorization of variables or merging of levels was performed. Hosmer- Lemeshow tests for goodness of fit were performed for model fitness^14^. The chi-square test was used to compare categorical variables between the groups. Bi-variable logistic regression analysis was performed to examine the determinant factors of outcome variables. For bivariable analysis, a P-value of ≤ 0.25 was used for multivariable logistic regression analysis. After checking for multicollinearity, a multivariable analysis was performed to adjust for possible confounders and identify significant determinant factors. Variables with a P-value < 0.05 were used to declare statistical significance in multivariable logistic regression analysis. The results are presented in text, tables, charts, and graphs.

### Data quality assurance

To ensure the quality of data, one day of training was provided for data collectors on the objectives and standard procedures of the data collection. The questionnaire was pretested on 5% of the sample before the actual data collection period. It was assessed for its clarity, length, and completeness, and the necessary corrections were made accordingly. The completeness of the data was evaluated daily by the principal investigator. Data collectors were supervised, and regular meetings were held throughout the data collection period.

### Ethics approval and consent to participate

Ethical approval was obtained from Nigist Eleni Mohamed Memorial Comprehensive Specialized Hospital (NEMMCSH) ethics committee (Ref.No.NEMMCSH/25/2022) to get permission to get access to charts of mothers for data collection. All methods were carried out in accordance with the principles of the Declaration of Helsinki. The requirement for informed consent was waived by the Ethics Committee of NEMMCSH (Ref.No.NEMMCSH/25/2022) because of the retrospective nature of the study.

## Results

In total, 140 controls and 70 cases of patient charts were enrolled in this study. The mean ± SD age of mothers of cases and controls were 26.9 ± 4.9 and 27.1 ± 4.1 years, respectively. Parents under the age of 25 years made up 64 (45.7%) of the control group and 32 (45.7%) of the case group. Of the parents of children included in this study, 29 (41.4%) of the cases and 85 (60.7%) of the controls of parents were urban residents (Table 2).

**Table 2:** Socio-demographic characteristics of mothers of a newborn with low and good Apgar score who gave birth by Cesaraen section.

Factors	Categories	Fifth-minute Apgar score		P*
		< 7	7–10	
Age (years)	< 25	32(45.7)	64(45.7)	0.87
	25–30	20(28.6)	36(25.7)	
	> 30	18(25.7)	40(28.6)	
Residence	Urban	29(41.4)	85(60.7)	0.08
	Rural	41(58.6)	55(39.3)	

*Pearson's chi-square test.

### Obstetric, Surgery and Anaesthesia-related characteristics

Among the total number of parents, 46 (65.7%) of parents of the cases and 94(67.1%) of parents of controls were multigravida. Regarding ANC follow-up, 97.1% of mothers in the case group and all mothers in the control group had ANC visits. In total, 62.9% of mothers in the case group and 54.6% of mothers in the control group underwent emergency surgery. This study has shown that 34(48.6%) of mothers of cases and 108(76.4%) of controls underwent Cesarean section with spinal anaesthesia. There was no significant difference in the proportion of mothers regarding the urgency of surgery between the case and control groups (P = 0.23) (Table 3).

**Table 3:** Obstetric characteristics of mothers of a newborn with low and good Apgar score who gave birth by Cesaraen section.

Factors	Categories	Fifth-minute Apgar score		Total	P*
		< 7	7–10		
Parity	Primigravida	24 (34.3)	46 (32.9)	70	0.83
	Multigravida	46 (65.7)	94 (67.1)	140	
ANC follow-up	Yes	68 (97.1)	140 (100)	208	0.16
	No	2 (2.9)	0	2	
Anemia	Yes	17 (24.3)	50 (35.7)	67	0.084
	No	53 (75.7)	90 (64.3)	143	
Type of Anesthesia	Spinal	36 (51.4)	108 (76.4)	144	0.43
	General	34 (48.6)	32 (23.6)	66	
Urgency of Surgery	Elective	26 (37.1)	65 (46.4)	91	0.23
	Emergency	44 (62.9)	75 (54.6)	119	
ID time	≤ 5 min	48 (68.6)	89 (63.6)	137	0.144
	> 5 mi	22 (31.4)	51 (36.4)	73	
Indication for Cesarean section	NRFHR( non-reassuring fetal heart rate )	6 (8.6)	19 (13.6)	25	0.45
	Scar	27 (38.6)	58 (41.4)	85	
	Pre/eclmpsia	12 (17.1)	12 (8.6)	24	
	Premature rupture of membrane (PROM)/ meconium	14 (20)	9 (6.4)	23	
	APH	2 (2.9)	10 (7.1)	12	
	Other (cord prolapse, breech, macrosomia, the prolonged second stage of labour, obstructed labour.)	9 (12.9)	32 (22.9)	41	

*CS* cesarean section, *APH* antepartum hemorrhage, *PROM* premature rupture of membrane, *NRFHR* non-reassuring fetal heart rate, *ID time* incision to delivery, *ANC* antenatal care.

*Pearson's chi-square test.

### Newborn Characteristics

Of the total, 24 (34.3%) of newborns in the case group and 22 (15.7%) newborns in the control group had low birth weight. All live newborns from the case group were admitted to the neonatal intensive care unit (NICU), whereas 97.1% of newborns from the control group were directly handed to the mother for early initiation of breastfeeding (Table 4).

**Table 4:** Newborns characteristics of mothers of a newborn with low and good Apgar scores who gave birth by Cesaraen section.

Factors	Categories	Fifth-minute Apgar score		Total	P*
		< 7	7–10		
Weight of newborn (g)	Low birth weight	24 (34.3)	22 (15.7)	46	0.001
	Normal birth weight	46 (65.7)	118 (84.3)	164	
The outcome of the newborn	Normal to mother	0	136 (97.1)	136	0.001
	NICU admission	67 (95.7)	4 (2.9)	71	
	Early neonatal death	3 (4.3)	0	3	

*NICU* Neonatal Intensive Care Unit.

*Pearson's chi-square test.

### Determinants of low fifth-minute Apgar score

Bivariable logistic regression analyses were conducted to identify determinants of low fifth-minute Apgar scores. The results revealed that urban residence (COR = 2.8, 95% CI: 1.58–5.9) P = 0.001, general anaesthesia (COR = 3.18, 95% CI: 1.72–5.82) P = 0.001), low birth weight of the newborn (COR = 2.78, 95% CI, 1.43‒5.4) P = 0.003, meconium-stained amniotic fluid (MSAF) (COR = 2.8, 95% CI, 1.01‒8.1) P = 0.05, were identified determinant factors.

Multivariable analysis was performed for the following variables; residence, type of anesthesia, weight of the newborn, type of surgery, and MSAF indication for cesarean section. According to the multivariable logistic regression analysis results, residence, type of anaesthesia, weight of the newborn, and type of surgery were identified as determinants of the fifth-minute low Apgar score.

The multivariable analysis result showed that the likelihood of developing a low Apgar score among neonates born to mothers who came from rural areas was 3.7 times (AOR = 3.7, 95% CI, 1.7‒8.1) higher compared with their counterparts. Moreover, neonates born with low birth weight were 3.2 times (AOR = 3.2, 95% CI, 1.3‒7.8) more likely to develop a low Apgar score than neonates with normal birth weight (Table 5).

**Table 5:** Determinant factors of a low Apgar score among newborns delivered by Cesarean section.

Variables	Categories	Fifth-minute Apgar score		COR	AOR	P
		** < 7 N (%)**	**7–10 N (%)**			
Residency	Urban	29 (41.4)	85 (60.7)	1	1	
	Rural	41 (58.6)	55 (39.3)	2.8 (1.5–5.1)	3.7 (1.7–8.1)	0.001*
Type of anaesthesia	Spinal	36 (51.4)	108 (76.4)	1	1	
	General	34 (48.6)	32 (23.6)	3.2 (1.7–5.8)	4.2 (1.9–9.3)	0.001*
Type of surgery	Elective	26 (37.1)	65 (46.4)	1	1	
	Emergency	44 (62.9)	75 (54.6)	1.4 (0.8–2.4)	2.6 (1.2–5.8)	0.015*
Weight of newborn	Low birth weight	24 (34.3)	22 (15.7)	2.8 (1.4–5.4)	3.2 (1.3–7.8)	0.012*
	Normal birth weight	46 (65.7)	118 (84.3)	1	1	
Indication	Scar	27 (38.6)	58 (41.4)	0.8 (0.28–2.4	0.9 (0.3–2.7)	0.99
	NRFHR	6 (8.6)	19 (13.6)	1.5 (0.5–4.36)	0.7 (0.2–2.2)	0.67
	APH	12 (17.1)	12 (8.6)	0.43 (0.8–2.2)	0.5 (0.15–1.9)	0.37
	Meconium	14 (20)	9 (6.4)	2.8 (1.01–8.1)	0.75 (0.9–8.5]	0.056
	Preeclampsia	2 (2.9)	10 (7.1)	0.8 (0.4–2.01)	0.2 (0.04–1.4)	0.122
	Other	9 (12.9)	32 (22.9)	1	1	

*COR* crude odds ratio, *AOR* adjusted odds ratios, *APH* antepartum haemorrhage, *NRFHR* non-reassuring fetal heart rate.

*Statistically significant.

Significant values are in bold.

## Discussion

This study included 210 (70 cases, 140 controls) singleton neonates delivered via Cesarean section; of which, 95.7% neonates from the case group were admitted to NICU and another three neonates from the same group had early neonatal death after being resuscitated. In the current study, factors that contributed to low fifth-minute Apgar scores among neonates delivered through the Cesarian section were determined. Newborns, delivered through emergency Cesarean section, with low birth weight (less than 2500gm), whose mothers are from the rural area, and delivered from mothers who undergone Cesarean section under general anaesthesia were identified as determinant factors in this study.

According to this study, neonates who were delivered with fifth-minute low Apgar scores were more likely from rural resident mothers than mothers from urban areas (AOR = 3.7, 95% CI, 1.7‒8.1). This result is supported by a hospital-based, unmatched case–control study conducted at Hawassa City Public Hospitals^6^. The results of a study conducted at the Gonder University referral hospital to assess the proportion and associated factors of low fifth-minute Apgar scores were consistent with our findings^12^. This might be related to low healthcare access in rural areas, which can directly affect fetal outcomes^15^. Lack of transportation, the distance of travel to the hospital by residents in rural areas, challenging roads, the cost of ambulance fuel, the use of an ambulance for unanticipated purposes, and the disobedient behaviour of ambulance drivers were additional concerns being evaluated^16,17^. Problem with referral pattern from primary health care will also have an impact on appropriate care of the mother.

This study revealed that neonates born with low birth weight ≤ 2.5 kg were 3.2 times more likely to have a low Agar score compared to neonates with normal birth weight (AOR = 3.2, 95% CI, 1.3‒7.8). This finding is in line with an unmatched case–control study conducted at Wolaita Sodo University Comprehensive Specialized Hospital^18^. Another hospital-based unmatched case–control study conducted to determine the determinants of birth asphyxia is consistent with our results^19^. This is because inadequate dietary intake, alcoholism, and passive tobacco smoking during pregnancy could affect neonatal birth weight, which could further affect Apgar scores^20^. Smaller newborns may experience difficult births, decreased temperature regulation, hypoglycemia, and may suffer challenging cardiorespiratory transition and perinatal hypoxia, all of which may further influence Apgar scores^21^.

The type of anaesthesia is another variable that has a significant association with a low fifth-minute Apagar score. Regarding our study findings, newborns whose mothers underwent general anaesthesia were 4.2 times more likely to develop a low fifth-minute Apgar score than those whose mothers underwent spinal anaesthesia. Our results are supported by those of multiple local and international studies. An institutional-based cross-sectional study conducted at Arba Minch General Hospital showed newborns delivered from a mother who underwent general anaesthesia were 4.39 times likely to develop low fifth-minute Apgar scores (AOR = 4.39, 95%CI = 2.45—7.88)^22^, case–control study conducted at Wolayta Sodo Comprehensive Specialized Hospital (WSCSH)^18^, and another study from Ghandi General hospital conducted to identify factors associated with fifth-minute low Apgar scores were consistent with our study findings^23^. This might be related to the high level of circulating catecholamines causing a reduction in uteroplacental blood flow and anaesthetic drugs administered during general anaesthesia cross the placenta and depress the fetus^24^.

The likelihood of low Apgar scores was approximately 2.6 times (AOR = 2.6;95% CI: 1.2–5.8) higher in neonates born from mothers who underwent emergency Cesarean section than in those born from mothers who underwent elective Cesarian section. This finding was supported by a study conducted to compare fetal outcomes in emergency versus elective Cesarean Section^25^ and another study that compared fetal outcomes after emergency and elective Cesarean section was also consistent with our results^26^. This might be because most of the mothers presented with complications or the decision to undergo Cesarean section might be delayed after they develop complications^27^.

### Limitations of the study

The authors could not obtain complete information about the economic status or the number of prenatal visits from the medical records of the mothers. Also, it was not possible to measure the urgency or severity of the emergency caesarean section because of the available data. All charts of mothers with term or post-term neonates who gave birth with Cesarian section were included in the study. However, the difference or similarity of the outcomes of term and post-term neonates could not be determined because of incomplete data recordings.

## Conclusions and recommendations

Emergency Cesarean section, newborns with low birth weight (less than 2500gm), mothers who came from rural areas, and mothers who had undergone Cesarean section under general anaesthesia were determinants of low fifth-minute Apgar score. Therefore, to limit the frequency of low fifth-minute Apgar scores and reduce complications, the indicated determinant factors must be addressed. Therefore, it is crucial to address the identified risk factors to minimize the incidence of low fifth-minute Apgar scores and reduce complications due to low Apgar scores. Particular emphasis should be given to mothers admitted from rural areas. Anaesthetists and the resuscitation team must take care when the procedure is an emergency and the preferred mode of anaesthesia is general. Careful identification of a fetus with low weight preoperatively would help the operating team undertake all required preparations.

### Supplementary Information

Supplementary Information.

## Data Availability

Data is provided within the manuscript or supplementary files.
